# Optimization of ultrasonically extracted β‐sitosterol from *Berberis jaeschkeana* using response surface methodology

**DOI:** 10.1002/fsn3.4215

**Published:** 2024-08-29

**Authors:** Awais Raza, Muhammad Nadeem Akhtar, Tahir Maqbool, Anees Ahmed Khalil, Mohamed Hassan Mohamed

**Affiliations:** ^1^ University Institute of Food Science and Technology, Faculty of Allied Health Sciences, The University of Lahore Lahore Pakistan; ^2^ University Institute of Diet and Nutritional Sciences, Faculty of Allied Health Sciences, The University of Lahore Lahore Pakistan; ^3^ Institute of Molecular Biology and Biotechnology, Faculty of Sciences, The University of Lahore Lahore Pakistan; ^4^ Department of Health Sciences, Faculty of Public & Environmental Health Somali International University (SIU) Mogadishu Somalia

**Keywords:** *Berberis jaeschkeana*, Box–Behnken design, response surface methodology, ultrasonic‐assisted extraction, β‐Sitosterol

## Abstract

*Berberis* genus is recognized as a significant source of β‐sitosterol and polyphenols. The inclusion of β‐sitosterol into various health‐related formulations has widened its potential in the pharmaceutical and nutraceutical industries. Process optimization ensures the maximum efficiency, consistency, and yield. In the current study, we employed mutual interaction effect to extract the β‐sitosterol from the root bark of *Berberis jaeschkeana* using ultrasonic‐assisted extraction (UAE) technique. In order to identify the optimal extraction parameters, we conducted a series of 29 experiments, varying factors, such as amplitude level, solid‐to‐liquid ratio, extraction time, and temperature. The mutual interaction effect encompasses several key components, including the Box–Behnken design, assessment of model fitness, coefficient of determination, analysis of variance (ANOVA), and the creation of three‐dimensional (3D) response curves of response surface methodology (RSM). The outcomes of the analysis revealed notable model fitness, highlighting the presence of linear, quadratic, and interactive effects among the various factors examined. The optimized UAE conditions (amplitude level of 30%, time of 10 min, solid‐to‐liquid ratio of 20, and temperature at 50°C) were applied. Under the most favorable extraction conditions, the β‐sitosterol was identified and quantified from *Berberis jaeschkeana* using high‐performance liquid chromatography (HPLC). The β‐sitosterol yield was measured at 43.52 mg per gram of the sample. Conclusively, the optimization approach for UAE using the mutual interaction effect contributes to a more rapid extraction process, resulting in a higher yield of β‐sitosterol. Furthermore, this study design could be extended to other valuable species or compounds to efficiently extract nutraceutical compounds and enhance the sustainable utilization of natural products.

## Graphical abstract

The ultrasonic‐assisted extraction (UAE) method was utilized in conjunction with mutual interaction effect to recover the important nutraceutical from *Berberis jaeschkeana*. We conducted a series of 29 trials, adjusting variables including temperature, sample‐to‐solvent ratio, extraction time, and ultrasonic power, to determine the ideal extraction parameters. The optimum parameters for ultrasonic‐assisted extraction (UAE) were executed, which included amplitude level 30%, time of 10 min, solid‐to‐liquid ratio of 20, and temperature of 50°C. For every gram of material, the production of β‐sitosterol was found to be 43.52 mg. High‐performance liquid chromatography (HPLC) was utilized to identify and quantify the β‐sitosterol from *B. jaeschkeana* under optimal extraction conditions.

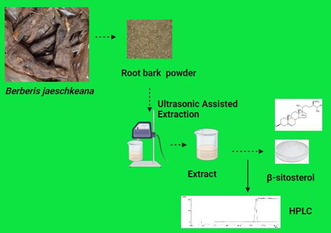

## 1 INTRODUCTION

*Berberis jaeschkeana* is indigenous to the Himalayan region, specifically to nations like India, Kashmir, Pakistan, Nepal, and Bhutan, and is a particular member of the genus *Berberis* (Khan et al., 2016). *B. jaeschkeana* is a deciduous one‐meter‐tall shrub with a stem exhibiting a faint reddish to yellow‐brown color (Alamzeb et al., 2015). Among species of *Berberis*, *B. jaeschkeana* is well known for its colorful berries, prickly branches, and occasionally its application in traditional medicine since it contains bioactive substances like β‐sitosterol (Alamzeb et al., 2013). The 10–15 mm long three‐cleft spines covered the shrub. The green leaves are 5–8 mm in width and 10–23 mm in length. Its flowers are 9–12 mm in range, and the blossoming is assembled in an umbellate or subumbellate manner (Andola et al., 2019). The β‐sitosterol is naturally found in plant sterol and is a member of the class of substances stated as plant sterols or phytosterols (Novotny et al., 2017). The plant sterols, especially the β‐sitosterol, have an extensive range of health benefits, and substantial health exploration has been done on them. The health benefits include antibacterial, anti‐arthritic, antidiabetic, antioxidant, hepatoprotective, and hypolipidemic effects (Parvez et al., 2018; Salehi et al., 2021). The structure of β‐sitosterol is almost similar to that of cholesterol and can be extracted from different plant genera like *Berberis, Urtica, Prunus, Nigella, Cucurbita, and Serenoa*. There are more than 500 species of the genus *Berberis* spreading all over the globe, and they are acknowledged for their substantial β‐sitosterol content (Khan et al., 2015). The β‐sitosterol is now spontaneously available in the market in a wide range of pharmaceutical and nutraceutical products. It has also been experimentally proved that β‐sitosterol has the capability to perform as an antioxidant by rummaging reactive oxygen species (ROS) and to activate antioxidant enzymes. Likewise, the use of β‐sitosterol is concomitant to lower the risk of cardiovascular disease development (Bin Sayeed & Ameen, 2015).

Many medical problems, including diabetes, cancer, Alzheimer's disease, and inflammation, have been effectively treated by these chemicals, which have been the subject of substantial research. The root barks of *Berberis* species are among the parts from which phytosterols can be isolated (Bakrim et al., 2022). The polyphenolics, phytosterols, and antioxidants were extracted from *Berberis jaeschkeana* under ideal ultrasonic‐assisted extraction (UAE) conditions and compared with conventional extraction (CE). Significant effects were seen in the stability of polyphenolics, phytosterols, and antioxidants and the yield as a function of solvent concentration, ultrasonic temperature, and sample‐to‐solvent ratio (Belwal et al., 2022). The extraction yield and extract quality are directly exaggerated by the bioprocessing parameters, which in turn affect the end product's cost. Conventionally, laboratory tests, including the UAE technique, were used to prepare *Berberis* root extract (Nuralın & Gürü, 2022). A categorization of steps, comprising the drying, extraction, and further separation and purification processes, are applied to acquire the required secondary metabolites or extract from plant resources (Anwar et al., 2020; Belwal et al., 2017). Reports have shown that the UAE approach has been applied extensively to extract a number of chemicals with medicinal and nutraceutical value. Compared to conventional extraction methods, the process generates surplus heat and pressure, which improves mass transfer and speeds up plant components’ extraction while producing higher yields (Santos et al., 2020). Excessive heat during UAE has been noted to potentially degrade heat‐sensitive compounds. Therefore, it is essential to optimize the processing conditions, including sonication power, time, temperature, and composition, to obtain a higher‐quality extract and preserve the integrity of the compounds (Belwal et al., 2022).

In the quest to establish the most favorable processing conditions, diverse attempts have been undertaken. Continual efforts are underway to explore novel techniques that can deliver β‐sitosterol of superior quality, possessing desirable attributes, all while maintaining cost‐effectiveness. Current research's primary focus has been to optimize the extraction process using ultrasonication and analyzing the yield of β‐sitosterol extracted from *Berberis jaeschkeana* root extract (BJRE).

## 2 MATERIALS AND METHODS

### 2.1 Preparation of *Berberis jaeschkeana* root powder (BJRP)

*Berberis jaeschkeana* roots and barks were harvested from their indigenous populations in Azad Kashmir during July 2023 and were identified by Prof. Dr. Rizwan Rasheed (Department of Botany, Government College University Faisalabad, Pakistan). A voucher specimen number GCUF‐BOT‐23‐3812 was deposited in the herbarium of Botany, Government College University Faisalabad. The collected samples underwent thorough washing and were subsequently air‐dried in the shade. The roots were then finely chopped and processed in a hammer mill, until they reached a particle size of less than 85 microns. The resulting root powder was carefully stored at 4°C in a refrigerator, and the extraction process was initiated within a 48‐h window after the grinding procedure.

### 2.2 Proximate analysis of BJRP

The moisture, crude protein, crude fat, total ash, and crude fiber contents of each sample were determined using standard methods from the Association of Official Analytical Chemists (AOAC). To ascertain moisture content, 2.0 g of each fresh sample was heated in a crucible in an oven held at a constant temperature of 105°C, until a constant weight was achieved. The remaining dry matter was utilized for determining the other parameters. Crude protein content (% total nitrogen × 6.25) was determined through the Kjeldahl method, employing 2.0 g samples. For crude fat determination, 5.0 g of the sample was exhaustively extracted in a Soxhlet apparatus using petroleum ether (boiling point range 40–60°C) as the extractant. Ash content was established by incinerating 10.0 g samples in a muffle furnace maintained at 550°C for 5 h. Crude fiber content was obtained by digesting 2.24 g of the sample with sulfuric acid (H_2_SO_4_) and sodium hydroxide (NaOH), followed by incinerating the residue in a muffle furnace that was also maintained at 550°C for 5 h. Moisture content was once again determined by heating 2.0 g of each sample to a constant weight in a crucible, placed within an oven maintained at 105°C (AOAC, 2019). All analyses were conducted in triplicate.

### 2.3 Ultrasound‐assisted extraction of BJRE from BJRP

Ultrasound (Model VCX 750, Sonics & Materials, Inc., Newtown, CT, USA) technology was employed to facilitate the extraction of BJRE from BJRP, using n‐hexane as the solvent. Using a digital weighing machine, the BJRP sample was precisely weighed (100 ± 0.1 g). To optimize the extraction process of BJRE from BJRP samples, we explored the effects of sonication amplitude level (%), sonication temperature (°C), the ratio of solid‐to‐liquid, and sonication duration (in min) using a Box–Behnken design (Tan et al., 2018). Table 1 represents the coded values of the experiment. A digital thermometer installed in an ultrasonic apparatus is used to regulate the temperature of the extracted solution within a narrow range of ±1.5°C. Following the completion of the extraction process, a 40‐mesh size screen was utilized for the filtration of the extracted solution. The Universal 320R, Hettich, Germany centrifuge machine was employed to separate the BJRE from the solution. The centrifugation was applied at 5000 rpm (revolutions per minute) for 10 min (Hryniewicka et al., 2020).

**TABLE 1:** The Box–Behnken design was employed to establish the coded and actual values of independent variables in order to optimize the yield of β‐sitosterol.

Sonication variables	Coded levels		
	−1	0	+1
Extraction temperature (°C)	50	70	90
Solid‐to‐liquid ratio	10	20	30
Amplitude level (%)	30	50	70
Sonication extraction time (min)	10	15	20

### 2.4 HPLC analysis of β‐sitosterol

High‐performance liquid chromatograph (HPLC) system with binary pumps (LC‐20AD) and a photodiode array detector were used to analyze β‐sitosterol. A C18 reverse‐phase column (100 × 2.1 mm inner diameter, 5 μm, Thermo Electron Corporation, HyPurity, Japan) maintained at a constant temperature of 40 ± 1°C was used to separate a 20 μL injection of the extract, which was made by dissolving 5 grams of dry extract in 20 mL of n‐hexane. The mobile phase was made up of a 90:10 ratio of methanol (A) and acetonitrile (B) with isocratic elution, which was pumped at a rate of 1.5 mL/min for the duration of the 20‐min run time. The lambda max was set at the detector wavelength of 202 nm (Khonsa et al., 2022).

### 2.5 Optimal UAE condition and validation of the model

The study aimed to find and validate the optimal UAE conditions for maximizing β‐sitosterol extraction while keeping all responses at their peak within the specified factor ranges. The model created optimal conditions based on response values, and a desirability criterion was applied to select the most favorable UAE conditions. The chosen optimal extraction conditions were subjected to a follow‐up experiment conducted in triplicate, and the coefficient of variation (CV) was calculated to validate the model (Guideline, 2005).

### 2.6 Statistical analysis

The Box–Behnken design was employed to model the behavior of a system, with a quadratic equation being utilized for this purpose. The level of significance of the β‐sitosterol yield was evaluated through data analysis performed using the Design‐Expert software package (Beckley et al., 2017). Three sonication runs were performed for each treatment, and the resulting average yields, along with their standard deviations, were used. A significance level of 5% was employed to determine the significant differences among the various treatments.

## 3 RESULTS AND DISCUSSION

### 3.1 Proximate analysis of BJRP

Proximate analysis of BJRP was important to explore its potential. Table 2 displays the mean compositional values of BJRP, revealing its abundant nutrient content, including protein (2.49% ± 0.68%), moisture (37.5 ± 0.56), fat (0.45% ± 0.38%), ash content (1.36% ± 0.23%), fiber (41.39% ± 0.43%), and carbohydrates (15.86% ± 0.66%). The earlier study showed relevant results of the composition of the fruits of the different varieties of *Berberis* (Batool et al., 2021).

**TABLE 2:** Chemical characterization of *Berberis jaeschkeana* root powder (BJRP).

Parameter	BJRP
Moisture (**%**)	37.5 ± 0.56
Ash (**%**)	1.36 ± 0.23
Crude fat (**%**)	0.45 ± 0.38
Crude protein (**%**)	2.49 ± 0.68
Crude fiber (**%**)	40.39 ± 0.43
Nitrogen‐free extract (NFE) (**%**)	15.86 ± 0.66

*Note*: The presented values indicate the mean ± standard deviation.

### 3.2 Optimization of ultrasonic‐assisted extraction for β‐sitosterol yield

The impact of various variables, including sonication extraction temperature (°C), the solid‐to‐liquid ratio, the amplitude level (%), and sonication extraction time (min), on the optimal extraction of β‐sitosterol was investigated using a Box–Behnken design with three levels and four factors. A total of 29 sonication extraction experiments were conducted, as specified by the Box–Behnken design. Table 3 presents the β‐sitosterol yields obtained from the different sonication extraction runs. The table clearly demonstrates that the β‐sitosterol yield ranged from 27.54 ± 0.21 to 43.20 ± 0.28 mg/g. This variation in the percentage of β‐sitosterol yield underscores the substantial influence of sonication extraction conditions on the yield of β‐sitosterol.

**TABLE 3:** Box–Behnken design for the yield of β‐sitosterol as response under UAE for different levels of sonication variables.

Sonication run	Sonication variables				Sonication response
	Amplitude level (%)	Solid/liquid ratio	Sonication temperature (°C)	Sonication time (min)	
					β‐Sitosterol (mg/g)
1	0 (50)	−1 (10)	−1 (50)	0 (15)	38.80 ± 0.78
2	0 (50)	−1 (10)	0 (70)	−1 (10)	33.22 ± 0.33
3	0 (50)	0 (20)	0 (70)	0 (15)	30.65 ± 0.51
4	1 (70)	0 (20)	−1 (50)	0 (15)	40.19 ± 0.42
5	0 (50)	0 (20)	0 (70)	0 (15)	30.70 ± 0.08
6	−1 (30)	0 (20)	1 (90)	0 (15)	28.45 ± 0.27
7	0 (50)	1 (30)	−1 (50)	0 (15)	37.65 ± 0.09
8	1 (70)	1 (30)	0 (70)	0 (15)	30.20 ± 0.22
9	1 (70)	0 (20)	0 (70)	−1 (10)	33.75 ± 0.18
10	0 (50)	1 (30)	1 (90)	0 (15)	28.75 ± 0.25
11	−1 (30)	0 (20)	0 (70)	−1 (10)	34.25 ± 0.19
12	0 (50)	−1 (10)	0 (70)	1 (20)	30.15 ± 0.17
13	−1 (30)	0 (20)	−1 (50)	0 (15)	41.15 ± 0.21
14	0 (50)	0 (20)	0 (70)	0 (15)	31.35 ± 0.28
15	0 (50)	0 (20)	−1 (50)	1 (20)	36.15 ± 0.14
16	0 (50)	0 (20)	0 (70)	0 (15)	31.40 ± 0.19
17	0 (50)	1 (30)	0 (70)	1 (20)	29.55 ± 0.18
18	−1 (30)	−1 (10)	0 (70)	0 (15)	32.45 ± 0.21
19	−1 (30)	0 (20)	0 (70)	1 (20)	29.80 ± 0.07
20	0 (50)	0 (20)	−1 (50)	−1 (10)	43.20 ± 0.28
21	0 (50)	−1 (10)	1 (90)	0 (15)	28.80 ± 0.19
22	−1 (30)	1 (30)	0 (70)	0 (15)	32.10 ± 0.15
23	0 (50)	0 (20)	0 (70)	0 (15)	31.59 ± 0.08
24	0 (50)	0 (20)	1 (90)	−1 (10)	29.29 ± 0.24
25	0 (50)	1 (30)	0 (70)	−1 (10)	34.29 ± 0.08
26	0 (50)	0 (20)	1 (90)	1 (20)	27.54 ± 0.21
27	1 (70)	−1 (10)	0 (70)	0 (15)	30.60 ± 0.48
28	1 (70)	0 (20)	0 (70)	1 (20)	33.95 ± 0.53
29	1 (70)	0 (20)	1 (90)	0 (15)	28.15 ± 0.55

### 3.3 Fitting the experimental model

Various indicators were utilized to assess the validity of the second‐order polynomial response model, encompassing measures, such as the coefficient of determination (*R* ^*2*^), CV, and the model's significance (*F*‐value). A regression model was utilized to estimate the predicted β‐sitosterol yield, and a comparison was conducted between these predicted values and the actual β‐sitosterol yield (Alam et al., 2020). R2 refers to the relationship between the described variations and the total variations (Akhtar et al., 2019). R2, the calculated value of the coefficient of determination, and adjusted R2, the adjusted coefficient of determination, were found to be 0.9635 and 0.9270, respectively. The response model's adequate precision of 18.7374 supports the validity and applicability of the applied second‐order polynomial model. The response model accurately anticipated 96.35% of the variation in the current experiment, as indicated by the calculated *R* ^*2*^ value for β‐sitosterol yields being near 1. The model would be consistent if the CV value were less than 5%, as shown by (Liu et al., 2022), which in the current study appeared to be accurate as the CV for β‐sitosterol production was 3.34%. This model finds its application to be used for the current experimentation based on the satisfactory findings provided by the current experimental ratio of 18.737.

The second‐order polynomial model equations obtained for β‐sitosterol yield in terms of coded variables are as follows:

$$\begin{array}{c} Y=+31.14-0.1133A-0.1233B-5.43C\\ \;-1.66D-0.0125\mathrm{AB}+0.1650\mathrm{AC}+1.16\mathrm{AD}\\ \;+0.2750\mathrm{BC}-0.4175\;\mathrm{BD}+1.08\mathrm{CD}+0.8348A^{2}\\ \;–0.2252B^{2}+2.34C^{2}+0.7222D^{2} \end{array}$$

where, A = Extraction temperature (°C). B = Solid to liquid. C = Amplitude level (%). D = Time.

Similarly, the second‐order polynomial model equations obtained for β‐sitosterol yield in terms of actual variables are as follows

$$\begin{array}{c} Y=31.13800–0.113333\;\text{Sonication}-0.123333\;\text{Ratio}-5.43000\;\text{Temp}\\ \;-1.65500\;\text{Time}-0.012500\;\text{Sonication}\;x\;\text{Ratio}+0.165000\;\text{Sonication}\times \text{Temp}\\ \;+1.16250\;\text{Sonication}\times \text{Time}+0.275000\;\text{Ratio}\times \text{Temp}\\ \;-0.417500\;\text{Ratio}\times \text{Time}+1.07500\;\text{Temp}\times \text{Time}\\ \;+0.834750\;\text{Sonicatio}n^{2}-0.225250\;\text{Rati}o^{2}+2.34475\;\mathrm{Tem}p^{2}+0.722250\;\mathrm{Tim}e^{2} \end{array}$$

where, Temp = Extraction temperature (°C). WMR = Solid‐to‐liquid ratio. Amp = Amplitude level (%).

The impacts of independent variables were assessed using the ANOVA as interaction, quadratic, linear, and residual coefficients. The results made it abundantly evident that model effects had a major impact on β‐sitosterol yield, as shown in (Table 4). When compared to interaction effects, the linear and quadratic effects displayed behavior that was very significant (*p* .01). For the extraction of β‐sitosterol yield from BJRE, the behavior of the various model effects was linear > quadratic > interaction. The table also made it abundantly evident that quadratic and linear coefficients were the most significant.

**TABLE 4:** Analysis of variance (ANOVA) for theβ‐sitosterol yield.

	Source	Sum of squares	Df	Mean square	F‐value	*p*‐value	
Linear	Model	439.25	14	31.37	26.41	.071	Non‐Significant
	A‐Sonication	0.1541	1	0.1541	0.1297	.03241	Significant
	B‐Ratio	0.1825	1	0.1825	0.1536	.7010	Non‐Significant
	C‐Temp	353.82	1	353.82	297.78	<.0001	Significant
	D‐Time	32.87	1	32.87	27.66	.0001	Significant
Interaction	AB	0.0006	1	0.0006	0.0005	.9820	Non‐Significant
	AC	0.1089	1	0.1089	0.0917	.7665	Non‐Significant
	AD	5.41	1	5.41	4.55	.0511	Non‐Significant
	BC	0.3025	1	0.3025	0.2546	.6217	Non‐Significant
	BD	0.6972	1	0.6972	0.5868	.4564	Non‐Significant
	CD	4.62	1	4.62	3.89	.0686	Non‐Significant
Quadratic	A^2^	4.52	1	4.52	3.80	.0714	Non‐Significant
	B^2^	0.3291	1	0.3291	0.2770	.6069	Non‐Significant
	C^2^	35.66	1	35.66	30.01	<.0001	Significant
	D^2^	3.38	1	3.38	2.85	.1136	Non‐Significant
	Residual	16.63	14	1.19			
	Lack of fit	15.89	10	1.59	8.50	.069	Non‐Significant
	Pure error	0.7479	4	0.1870			
	Cor Total	455.88	28				

*Note*: R^2^: 0.9635. Adjusted R^2^: 0.9270. Predicted R^2^: 0.7967. Adequate precision: 18.7374.

Abbreviation: Df, degree of freedom.

### 3.4 Single factor analysis

The impact of various sonication extraction parameters, including sonication temperature (°C), the solid‐to‐liquid ratio, sonication amplitude level (%), and the duration of sonication (in minutes), was assessed by adjusting these factors within a range of −1 to +1 for each extraction variable to determine their effect on the yield of β‐sitosterol. The temperature during sonication (°C) exhibited a favorable influence on the extraction of β‐sitosterol. When all other extraction parameters were held constant at their average values, an upward trend in β‐sitosterol yield was evident with higher sonication temperatures (°C). But at higher temperatures at 90°C, the decrease in the yield was noticed. Elevating the temperature during ultrasound‐assisted extraction causes an increase in vapor pressure, subsequently reducing the cavitation force and ultimately resulting in a decreased yield. These findings distinctly indicate the substantial impact of sonication temperature (°C) on the extraction process (Zhang et al., 2017).

Also, at a higher amplitude level (%), when all other variables were maintained at their average values, the generation of free radicals resulted in a decreased yield (Dzah et al., 2020). Due to the preference for mass transfer, the ratio of liquid to solid from 10 to 30 showed an increasing trend in the yield of β‐sitosterol (Santos et al., 2020). Likewise, there is a limitation in the solid‐to‐liquid ratio, reaching a maximum of 20, as exceeding this threshold does not significantly enhance the yield of β‐sitosterol. It can have adverse effects on the functional properties of the extract, thereby restricting its potential applications in the food industry (Feng et al., 2014). The sonication extraction time (in minutes) also displayed a significant impact when all other factors were maintained within their average ranges. There was a decrease in the yield of β‐sitosterol with the increase in time due to excessive cavitation‐induced milling of plant particles and the breakdown of β‐sitosterol (Hu et al., 2022).

### 3.5 Analysis of mutual interaction effect

Response surface methodology (RSM) was employed to optimize the extraction conditions for β‐sitosterol, which encompassed variables, such as sonication extraction temperature (°C), solid‐to‐liquid ratio, amplitude level (%), and sonication extraction time (in minutes). Estimating the β‐sitosterol yield proved challenging when altering interdependent responses using the response model. The highest β‐sitosterol yield was achieved by manipulating the responses of two extraction variables while maintaining the other two variables at their mean coded values.

**FIGURE 1:**
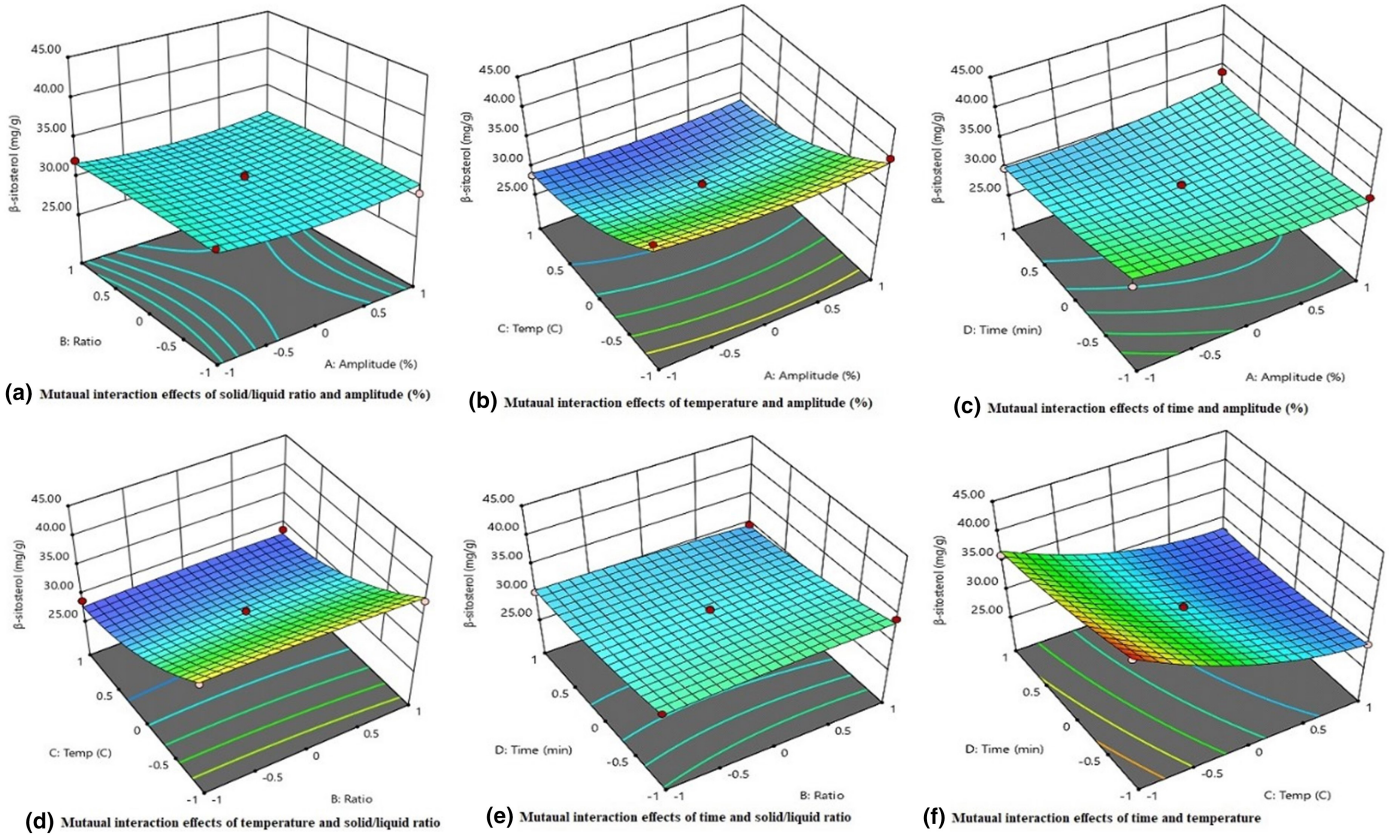
Effect of mutual interaction of independent variables on β‐sitosterol yield.

The mutual impact of amplitude level (%) and the ratio of solid to liquid showed that the yield of β‐sitosterol improved at the lower levels of independent variables. These findings have been visually depicted in Figure 1a. When examining the interaction between sonication extraction temperatures (°C) and amplitude level (%) while maintaining all other variables at their average levels, a noticeable decline in the yield of β‐sitosterol was observed with the increase of temperature and amplitude level (%), as shown in Figure 1b. The yield of β‐sitosterol decreased at the high levels of amplitude level (%) and time (in minutes) interaction (Figure 1c). It has also been observed that increasing the sonication temperature and the ratio of solid to liquid decreased yield of β‐sitosterol (Figure 1d). Furthermore, the results showed that the β‐sitosterol yield increased the sonication time and solid‐to‐liquid ratio at a lower mutual interaction level, as shown in Figure 1e. The yield of β‐sitosterol ranged from 27.54 to 43.20 mg/g, as the sonication extraction temperature and time varied between −1 and +1. By varying these two variables while maintaining the other two at their mean values, we observed the lowest and highest yields of β‐sitosterol (Figure 1f). When investigating the combined influence of sonication temperature and extraction time, we noticed improved yield of β‐sitosterol at decreasing temperature and time. There are limited published data available that support our findings regarding the yield of β‐sitosterol through the use of RSM.

The current study's findings regarding the extraction of β‐sitosterol from sugarcane rind align with prior research conducted using particular extraction conditions that impact the yield of β‐sitosterol from this source. The optimized extraction conditions were determined as follows: a liquid‐to‐solid ratio of 7.94 mL/g, an extraction temperature of 50°C, and an extraction time of 5.98 h (Feng et al., 2014). In a related study, the influence of various independent factors, including extraction temperature (ranging from 25 to 65°C), extraction time (ranging from 15 to 45 min), and the ratio of liquid to solid (varying from 60% to 90%), on the dependent variable, namely the yield of β‐sitosterol, was examined using RSM. The study concluded that the optimal conditions for obtaining the highest extract yield (218.82 mg/g) were achieved at a temperature of 43°C, an extraction time of 32 min, and a solid‐to‐liquid ratio of 80% (Ullah et al., 2023).

Prior research and our current experiments have indicated that increased pressure and longer extraction time result in higher yields of walnut oils. However, the temperature has a negative impact at lower pressures and a positive effect at higher pressures. The application of shorter extraction times and moderate pressure provides extracted fractions enriched in unsaturated fatty acids, as a result yielding refined oils with raised levels of nutritional and health‐promoting ingredients (De Zordi et al., 2017). Additionally, research has revealed that the yield of β‐sitosterol was significantly exaggerated by the interaction between time and temperature (Li et al., 2015). Conversely, when keeping a constant temperature (in degrees Celsius) and duration (in minutes), the progressive rise in β‐sitosterol yield can be credited to variations in the liquid‐to‐solid ratio. These findings may be attributed to the extra liquid causing the exclusion of gums from the *Desmostachya bipinnata* extract (Subramaniam et al., 2015).

The prior findings and the present research results guided the model circumstances for succeeding in a high β‐sitosterol yield were determined to be the ratio of liquid to solid of up to 20 and extraction temperature of 80°C. These particular parameters led to an excessive yield of almost 10% relative to the dry weight of the meal. Using a Box–Behnken design in the context of RSM in combination with an HPLC method that has been validated illustrates great potential as a greatly effective method for both quantitatively measuring the β‐sitosterol and exploiting the extraction parameters that are extracted from *Astragalus atropilosus* (Alam et al., 2020).

### 3.6 Validation of optimal UAE condition

The optimal UAE conditions for extracting β‐sitosterol from *B. jaeschkeana* were determined by selecting the condition with the highest desirability score from the model. The chosen optimal conditions involve using a 30% amplitude level, a solid‐to‐liquid ratio of 20, and 10‐min extraction duration at a temperature of 50°C. The obtained response values were very close to the predicted values from the model. Under this condition, β‐sitosterol content was found to be 43.52 mg/g, as shown in (Table 5).

**TABLE 5:** Validation of optimal UAE condition for β‐sitosterol in *B. jaeschkeana* roots.

Sonication variables				Predicted value of β‐sitosterol (mg/g)	Experimental value of β‐sitosterol (mg/g)	Residual error (%)
Amplitude level (%)	Liquid/solid ratio	Sonication temperature (°C)	Sonication time (min)			
30	20	50	10	44.64	43.52	2.57

## 4 CONCLUSION

An improved ultrasonic‐assisted extraction method for extracting the β‐sitosterol from *Berberis* species has been successfully developed and validated. Under the optimized UAE conditions, the concentration of β‐sitosterol (43.52 mg/g) extracted from *B. jaeschkeana* root bark was notably higher, compared to previously reported concentrations. As regards the widespread commercial use of β‐sitosterol in a multitude of pharmaceutical and nutraceutical products, our current study offers a dependable, reproducible, and cost‐effective UAE method. This method has demonstrated enhanced extraction yields, making it a valuable contribution to the food industry. Therefore, the findings of this study can serve as a model for replication in other high‐value medicinal plants for effective and maximum extraction of nutraceutical components and sustainable utilization.

## AUTHOR CONTRIBUTIONS

**Awais Raza:** Writing – original draft (equal). **Muhammad Nadeem Akhtar:** Methodology (equal); writing – review and editing (equal). **Tahir Maqbool:** Formal analysis (equal). **Anees Ahmed Khalil:** Investigation (equal). **Mohamed Hassan Mohamed:** Supervision (equal).

## CONFLICT OF INTEREST STATEMENT

No potential conflict of interest was reported by the author(s).

## CONSENT FOR PUBLICATION

All authors agree to publish.

## Data Availability

The authors confirm that data supporting the findings of this study are available within the article.
